# Important Treatment Outcomes for Patients with Psoriatic Arthritis: A Multisite Qualitative Study

**DOI:** 10.1007/s40271-017-0221-4

**Published:** 2017-02-22

**Authors:** Emma Dures, Sarah Hewlett, Jane Lord, Clive Bowen, Neil McHugh, For the PROMPT Study Group, William Tillett

**Affiliations:** 1 University of the West of England, Bristol, UK; 2 University Hospitals Bristol, Bristol, UK; 3 The Psoriatic Arthritis Support Group (PsAZZ), Bath, UK; 4 University of Bath, Bath, UK; 5 Royal National Hospital for Rheumatic Diseases, Bath, UK; 6 Academic Rheumatology, Bristol Royal Infirmary, Bristol, BS2 8HW UK

## Abstract

**Background:**

Psoriatic arthritis (PsA) is a variable and complex inflammatory condition. Symptoms can compromise physical function, reduce quality of life, and accrue significant health costs. Commonly used patient-reported outcomes largely reflect the professionals’ perspective, however it is not known whether they capture what is important to patients.

**Objective:**

The aim of our study was to identify treatment outcomes important to patients with PsA.

**Methods:**

Eight focus groups that were audio recorded, transcribed, anonymised and analysed using inductive thematic analysis were conducted at five hospital sites. The full data set was analysed by the lead researcher, and subsets analysed by three team members (including patient partners).

**Results:**

Overall, 41 patients sampled for a range of phenotypes and domains of disease activity participated in the study: 20 males; mean age 58 years (range 28–75, standard deviation [SD] 11.4); mean disease duration 9 years (range 0.5–39, SD 8.3); and mean Health Assessment Questionnaire score of 1 (range 0.0–2.5, SD 0.7). Over 60 outcomes were identified and grouped into four themes: (i) symptom alleviation (e.g. pain, fatigue, itchy skin, swelling, and reducing variability); (ii) reduction of disease impact (e.g. tiredness and pain, mobility and dexterity, deteriorating physical fitness, negative emotional responses, and strained relationships and social interactions); (iii) improved prognosis (e.g. slowing down disease progression, maintaining independence, and enhancing quality of life); and (iv) minimisation of treatment harm and burden (e.g. nausea, long-term effects, and administration and monitoring of treatments).

**Conclusions:**

Outcomes from treatments that are important to patients, which relate to impacts from PsA and its treatment that range beyond those outcomes commonly measured, were identified. These patient perspectives need to be considered when evaluating treatments.

## Key Points for Decision Makers

**Table:** 

Patients identified important outcomes beyond those that are commonly evaluated.
There is a need to establish how identified outcomes are represented in existing measures.
The outcomes identified reflect patients’ treatment beliefs and influence their treatment decisions.

## Background

Psoriatic arthritis (PsA) is a complex inflammatory condition, comprising five phenotypes: polyarthritis, oligoarthritis, axial, distal interphalangeal, and mutilans. Symptoms can include a red, scaly rash (psoriasis), inflammation of the tendons and ligaments (enthesitis), swelling in the fingers and toes (dactylitis), stiff and painful joints, thickening and pitting of the nails, and fatigue, and can impair physical function, cause disability, and reduce quality of life [1]. In addition, PsA can accrue significant health costs. For example, up to half of patients with PsA have some level of work disability, and three in ten are unemployed [2].

PsA is estimated to affect 19/10,000 people in the UK [3]. Among people with psoriasis, this increases to approximately 10%, with higher prevalence in those with more extensive skin disease [4]. Many treatments are available for the management of PsA, including methotrexate and other conventional synthetic disease-modifying antirheumatic drugs (csDMARDs), as well as numerous biological DMARDs (bDMARDs) [5]. The recommended treatment target is remission or, alternatively, low disease activity [6]. However, response to treatments varies across the different manifestations of PsA, highlighting that it is not a clinically or therapeutically homogeneous disease [7].

Many outcomes reported in relation to disease activity in PsA reflect clinicians’ and researchers’ views about domains which should be assessed. Moreover, the patient-reported outcome measures (PROMs) used to capture them have typically been designed without significant input from patients [8–10]. This is counter to recommendations and means that research and clinical practice might fail to measure outcomes that matter to patients [11]. As an example, the patient-reported core domains for PsA were peripheral joint activity, skin activity, pain, patient global assessment, physical function, and health-related quality of life. Only four patients contributed to this when it was proposed at the Outcome Measures in Rheumatology (OMERACT) Conference, therefore consensus was derived largely from professional views, and OMERACT requested further patient input [12, 13].

A PROM designed to capture PsA impact is the Psoriatic Arthritis Impact of Disease (PsAID) questionnaire, the development of which involved 12 patient research partners from European countries who discussed the findings of a literature review examining existing PROMs that might capture impact. This was followed by a ranking exercise and validation study with patients [14]. Although the PsAID includes a patient perspective, the domains within it were developed from the review of existing PROMs driven by clinicians’ perspectives.

It is crucial that treatment trials measure outcomes that are meaningful to patients. Understanding what patients want and expect from treatment also has implications for clinical practice. The aim of our study was to capture the perspective of patients with PsA with regard to important treatment outcomes. The study findings are reported in accordance with the Consolidated Criteria for Reporting Qualitative Research guidelines [15].

## Methods and Patients

This study was approved by the National Research Ethics Service Committee North West-Haydock (reference 15/NW/0609). Qualitative methods were used as the study aimed to explore patients’ experiences and views. Focus groups were selected for data collection because they facilitate debate and clarify convergent and divergent views among those taking part [16]. The focus group discussions were based on a topic guide designed by the research team, comprising patient partners (JL, CB), rheumatology clinical academics (WT, SH, NMH), and a qualitative researcher (ED) (Table 1).

**Table 1:** Topic guide

Key questions in focus groups
Which symptoms have the most effect on your well-being?
What do you want from your treatment?
What are the benefits and drawbacks of treatment for you personally?
How do you know when you are in a flare?

The research team designed a maximum variation sampling frame to guide their recruitment strategy and include patients with a range of age, disease duration, sex, PsA phenotypes and domains of disease activity. Patients were eligible to take part if they were over 18 years of age with a clinician-confirmed diagnosis of PsA, had sufficient English language to participate in discussions, had capacity to give informed consent, and fulfilled the sampling frame criteria. Recruitment was conducted at each site by local members of the rheumatology research team. Eligible patients were either given a study information pack when they attended a hospital outpatient clinic, or a study information pack was sent to them in the mail. Study information packs included a cover letter from a clinician in the local rheumatology team and a patient information sheet. Prior to the start of the focus groups, patients provided written consent, demographic data (age, sex, disease duration), information about current medications and levels of disability (Health Assessment Questionnaire [HAQ]) [17] and answered the question ‘Are you experiencing a flare of your psoriatic arthritis today?’, using the response options ‘yes’, ‘no’ or ‘not sure’.

Focus groups comprising four to seven participants were conducted in nonclinical rooms at five hospital sites in England. They lasted between 63 and 87 min and were cofacilitated by ED and SH, both experienced qualitative researchers. Focus groups were audio recorded and transcribed, with additional note-taking to aid transcription. Transcripts were anonymised by replacing participants’ names with pseudonyms and removing all place names. Data were analysed manually using inductive thematic analysis [18]. First, data were coded by reading transcripts multiple times and making notes of words or short phrases that captured what was being said in the focus groups. Next, lists of words and short phrases from transcripts were reduced by removing duplications. Conceptually-related codes were then grouped together to inform overarching themes and subthemes. This data-driven approach was used because no ‘a priori’ theories were applied to the data. The full data set was analysed by ED, with a subset analysed independently by JL, CB and SH. Findings were shared, discussed and agreed by coauthors. Thematic saturation (no new information emerging) was achieved within six focus groups, with the final two focus groups being confirmatory [19].

## Findings

Overall, 41 patients took part in eight focus groups: 20 males; mean age 58 years (range 28–75, standard deviation [SD] 11.4); mean disease duration 9 years (range 0.5–39, SD 8.3); and mean HAQ of 1 (range 0.0–2.5, SD 0.7). Thirteen participants reported being ‘in flare’ (not sure = 5), and the sample included a range of phenotypes and domains of disease activity (Table 2).

**Table 2:** Sampling framework: number of participants with characteristic types of PsA and affected parts of the body

*PsA phenotypes*				
Polyarthritis (30)	Oligoarthritis (9)	Distal interphalangeal (18)	Axial (8)	Mutilans (1)
*Domains of disease activity*				
Skin (34)	Joints (36)	Spine (7)	Eye (2)	Enthesitis^a^ (14)

*PsA* psoriatic arthritis

^a^Inflammation at tendon, ligament or joint capsule insertions

Sixty-three important outcomes from treatment were identified, ranging from specific (e.g. difficulty with grip) to overarching (e.g. well-being); however, it should be noted that these were not 63 distinct outcomes. The range in the scale and specificity of the outcomes meant that some were conceptually related or overlapping, for example pain affecting specific parts of the body and being in pain generally. These 63 outcomes were grouped into 18 subthemes, then four main themes (Table 3).

**Table 3:** Main themes and subthemes

Main theme	Subtheme
Symptom alleviation	Pain throughout the body Physical and mental fatigue Itching, flaking skin Inflammation, swelling and stiffness Reducing variability
Reduction of disease impact	Overwhelming tiredness and pain Limited mobility and dexterity Deteriorating physical fitness Poor quality and disrupted sleep Negative emotional responses Strained relationships and social interactions
Improved prognosis	Slowing down or halting disease progression Enabling independence to be maintained Enhancing quality of life, well-being and sense of normality
Minimisation of harm and burden	Nausea and sickness Concerns about long-term effects Modes of administration Monitoring requirements

Findings are evidenced with data excerpts, followed by participant ID, age (in years), and focus group number.

### Theme 1: Symptom Alleviation

The alleviation or reduction of the physical symptoms experienced on a regular basis was one of the most important outcomes of treatment for participants.

*Pain throughout the body* Participants described pain in their joints (including hands, wrists, feet, hips and knees), their muscles (in particular shoulders) and their back. They also discussed painful tenderness, for example in tendons at the back of the foot.

*“I get pain in various joints round my body at different times” [Dave, 71, FG5]*

*“I seem to have it [pain] all over my body, mainly it’s my feet, my knees, legs” [Mel, 52, FG6]*

While some participants found that treatments controlled their pain, others always had some level of pain present.

*“I’m always in pain it’s just how much” [Mark, 28, FG1]*

*Physical and mental fatigue* Fatigue emerged clearly as a symptom that patients found a challenge to manage. Physical exhaustion was a major component, but some patients also experienced mental and emotional fatigue.

*“The most important thing is tiredness, just feeling I can sleep at any time” [Louise, 48, FG5]*

*“It’s as if your brain’s fatigued, you know, it’s as if it’s something else, it’s not just tired, it’s beyond that” [Judith, 65, FG8]*

*“I feel so drained, so washed out, I can’t be bothered” [Ameila, 75, FG8]*

Several participants identified fatigue was particularly important because it was not alleviated by their current treatments.

*“The more the disease is controlled by the drugs, the more the fatigue is a problem” [Adam, 41, FG3]*

*Itching, flaking skin:* For participants with visible psoriasis, the symptom was associated with discomfort and embarrassment.

*“The psoriasis side of it is a big part for me … it’s not even really the response of other people it’s your perception of it …*

*Yeah exactly …*

*You feel horrible and scabby” [Paul, 50; Andrew, 72; and Miriam, 50, FG4]*

Skin improvement was an important treatment target, with several participants explaining that their PsA medications effectively controlled their psoriasis.

*“It was itchy and unsightly but not all that long after I started the medication it disappeared” [Natalie, 68, FG1]*

*Inflammation, swelling and stiffness* Participants highlighted inflamed, swollen and stiff joints as debilitating and therefore important to address through treatment.

*“Inflammation just generally runs your body down anyway, your body constantly fighting and stuff” [Duncan, 56, FG6]*

*“It’s these two joints [in hands] that are the worst and they’ve just become very swollen, incredibly tender, very stiff” [Alison, 66, FG5]*

*Reducing variability* Flares of disease activity were practically and psychologically challenging. In George’s case, he was not convinced that treatment was having a beneficial effect on his PsA until he stopped taking it and his symptoms returned. For Nicky, the sudden, aggressive flare of disease activity was alarming. Consequently, reducing symptom variability became important alongside reducing severity.

*“I had to leave the medication off for about three months and then I realised the medication was working because then the flare ups began” [George, 70, FG2]*

*“Overnight it was just out of control and I did manage to get an appointment and had bloods taken and then my inflammation had just gone sky high” [Nicky, 50, FG4]*

### Theme 2: Reduction of Disease Impact

While participants’ experiences of symptoms and taking treatments varied, there was widespread agreement that an important outcome was to reduce the impact of PsA on their daily lives.

*Overwhelming tiredness and pain* Reducing disease impact was closely related to symptom alleviation. Fatigue and pain caused a major impact on daily life and mood if they were not controlled.

*“You’re continuously drained during the day, and you can’t concentrate on whatever you’re trying to do, whether it’s driving, working, walking, anything” [Siddiq,39, FG6]*

*“The pain and the consequences of the pain in terms of immobility, in terms of moods and depressions, and feeling low and so on” [Andrew, 72, FG4]*

*Limited mobility and dexterity* A reduction in physical functioning, such as walking and strength and precision of grip, could have a significant impact. Restoring mobility and dexterity were therefore important treatment outcomes.

*“It’s lack of mobility that affected me and standing out therefore in the work place, having people stop at the bottom of the stairs to let you up or down and just not being normal” [Miles, 61, FG8]*

*“I have it in my thumb, which is annoying, because I’m an artist, and when it’s stiff I get frustrated because I can’t quite do what I used to” [Claire, 44, FG3]*

*Deteriorating physical fitness* Among the losses discussed were physical fitness and enjoyment of sport and exercise. Closely related was the unwanted consequence of weight gain.

*“I was quite a fitness freak, I used to go running, go to the gym, I had a very, very active life. I miss that” [Kate, 61, FG2]*

*“When I’m not well I can’t cycle and then I start putting on weight” [Janet, 65, FG1]*

*Poor-quality and disrupted sleep* Participants described how joint pain and stiffness impacted on their quality of sleep, with many unable to find effective treatments.

*“The discomfort because that hip, that shoulder, my back, and you see you just don’t have a good night’s sleep, ever” [Joanna, 57, FG4]*

*“Nothing seems to work, so lack of sleep is becoming vital now” [Sue, 75, FG5]*

*Negative emotional responses* The consequences of living with PsA, characterised by pain and fatigue and requiring ongoing management, could evoke negative emotional responses, including low mood, depression, anger and frustration.

*“I’m treated for anxiety and depression as well because of lack of sleep basically and constantly being in pain and run down” [Justin, 44, FG6]*

*“I do feel like it’s affected my emotion, I’ve become quite angry and resentful” [Abby, 41, FG7]*

*Strained relationships and social interactions* A perceived lack of understanding and unrealistic expectations of others in relation to their PsA could put a strain on participants’ relationships and limit their social interaction.

*“I have to constantly explain to my work, my wife, my children, my family, my friends why I’m not going out, why I’m not doing this, and so yeah, that makes me feel quite, the emotional side of that makes me quite insular” [Mark, 28, FG1]*

*“Mentally it’s massive and I find it’s hard to get other people to recognise it as well … I was in a marriage for 21 years and it was a big effect on that marriage” [Stephen, 43, FG3]*

### Theme 3: Improved Prognosis

In addition to focusing on alleviating symptoms and their impact in the immediate- and short-term, participants discussed the importance of treatment providing an improved prognosis in the medium- and long-term.

*Slowing down or halting disease progression* A factor influencing many participants’ treatment decisions was the potential for medications to slow down or halt future joint damage. This was important for those participants who expressed anxiety about their PsA worsening over time.

*“They said it will stop your disease activity so it won’t, your bones won’t fall to bits effectively” [Miriam, 50, FG4]*

*“The worry is always there that this is going to get worse and worse” [Alison, 66, FG5]*

*Enabling independence to be maintained* Increased disability in the future concerned those participants who placed a high value on maintaining their independence.

*“If I could have anything it would be independence, it would be to be able to be as fast as everybody else, it will be able to drive my own car, go out when I wanted to go out, come in and lock my own front door and not have somebody to come in to help with the shower” [Judith, 67, FG8]*

*“I live alone and I want to keep my independence” [Flora, 59, FG7]*

*Enhancing quality of life, well-being and sense of normality* While participants identified specific aspects of PsA that currently affected them, there were also overarching outcomes that were meaningful and potentially long-term, for example ‘well-being’. Participants consider these outcomes as they *“seek normality” [Paul, 50, FG4].* For Kate, the perceived risks of treatments were outweighed by the opportunity they offered to have an acceptable quality of life.

*“I have got a shorter life because of the amount of drugs that I take, I know that it is going to restrict my lifestyle. I have always said I want a good quality even though it is short; I don’t want to live until I am 90 and be curled up in a ball somewhere, I don’t want that, I would rather keep taking the injections and keep going [Kate, 61, FG2]*

### Theme 4: Minimisation of Treatment Harm and Burden

Some participants described their pharmacological treatments as ‘miraculous’, yet there were also high levels of anxiety. Beliefs about the balance between potential benefits of controlling disease activity and joint damage with potential harm from taking medications over time influenced patients’ priorities and treatment decisions.

*Nausea and sickness* Participants frequently described experiencing unpleasant side effects such as nausea and sickness in relation to their treatments.

*“It [methotrexate] just made me feel so dreadful and I had every side effect … just couldn’t tolerate it … nausea and just everything, it was awful.*

*I had much the same experience with methotrexate, I just felt dreadful all day, sick, general loss of appetite, lethargic” [Louise, 48, and Dave, 71, FG5]*

While Dave was not taking any pharmacological treatments at the time of the focus group as a consequence of side effects, Louise had gone on to try Humira^®^, which she was tolerating well (although it was not alleviating her troublesome fatigue). Another side effect that caused anxiety was lowered immunity to infection. Stuart described *“resisting*” DMARDs and taking Naproxen only.

*“What the methotrexate can do, it can affect your immune system down a bit, and I feel I could be undoing all the good that they’re trying to do at haematology by taking it” [Stuart, 60, FG7].*

*Concerns about long-term effects* Some participants expressed concerns about the long-term effects and possible toxicity of pharmacological treatments, and believed that medication might cause more damage than their PsA.

*“Just the thought of taking more medication and taking that long term, that bit worries me” [Claire, 44, FG3]*

*“I think you could be doing yourself more harm than good at times by taking these drugs” [Michael, 69, FG5]*

*Modes of administration* Practical difficulties in relation to self-injecting, getting tablets out of packaging or bottles, and swallowing large numbers of tablets or large-sized tablets were identified as barriers to taking medicines.

*“I really don’t want to do my injection, or when I was taking the tablets, I don’t really want to gag” [Janet, 65, FG1]*

*Monitoring requirements* The burden of adhering to monitoring requirements was too much for some participants, who found accessing services at the appropriate time incompatible with other commitments.

*“Access to the monitor side of it was part of the reason I stopped [treatment] because it didn’t suit my personal circumstances” [Chris, 44, FG2]*

## Discussion

The primary concern of participants was the ability of treatments to alleviate symptoms and, in turn, reduce the negative impact of disease. Pain was an unsurprising outcome and one that is widely measured [20]; however, fatigue and its impact featured heavily in discussions. Although fatigue is increasingly recognised as a symptom of PsA, it is not routinely addressed in either research or clinical practice. Participants’ accounts of the ineffectiveness of some treatments to ameliorate fatigue highlight the potential for nonpharmacological approaches. In rheumatoid arthritis, for example, a randomised controlled trial based on cognitive behavioural therapy was shown to effectively reduce the impact of fatigue [21]. If patients evaluate their treatment success on such outcomes, then unless professionals measure these, there is the potential for a mismatch as to how ‘treatment success’ is defined, which might affect decisions on treatment escalation or discontinuation. Understanding patient values will also help clinicians and researchers target specific issues that are undertreated.

These data provide insight into experiences and views likely to influence patients’ treatment decisions. They support evidence that nonadherence is consistently associated with psychological factors (including greater treatment concerns, lower treatment self-efficacy [i.e. confidence in one’s ability to follow treatment] and depression) and contextual factors (including practical barriers and a suboptimal patient–clinician relationship), many of which are modifiable risk factors [22]. Early diagnosis of PsA, management of disease progression, and management of impact through patient involvement in management plans are areas of clinical care identified as requiring improvement [23]. Our study findings strongly support this.

Some of these outcomes, for example those relating to adverse effects and drug safety, are routinely measured and will continue to be so. In addition to the interrelated and overlapping nature of some outcomes, these data present conceptual and measurement challenges. This includes the difficulty of distinguishing between symptoms and their impact and unpicking cause and effect; for example, it is possible that negative emotional responses were a symptom of high circulating levels of inflammatory cytokines associated with active PsA which are known to induce depressive-like behaviours, rather than a response to pain. However, the guiding principle when grouping outcomes into themes was to present patients’ experiences and beliefs. In taking this approach, we found that our data support the concept of the impact triad when considering the implications for measurement. The impact triad proposes that we need to capture severity, personal importance and self-management of symptoms to characterise the personal life impact of rheumatic diseases [24]. One example of measures that have done this is the Bristol Rheumatoid Arthritis Fatigue Scales (BRAFs) [25, 26]. These include the multidimensional BRAF-MDQ, which captures Living with Fatigue, Physical Fatigue, Emotional fatigue, and Cognitive Fatigue; and three BRAF Numerical rating Scales (BRAF NRS), which capture Severity, Coping, and Effect. The ability to measure patients’ experiences of fatigue and its impact in this way is potentially important for understanding individual responses and tailoring interventions and treatment.

Another conceptual and measurement challenge is the variation in the language used (e.g. participants’ own words), which might reflect different ways of expressing similar outcomes. At this stage, it was important to stay close to participants’ data to identify important outcomes and minimise imposing the research team’s interpretation beyond grouping related outcomes as part of the inductive analysis. However, it is neither feasible nor desirable to measure all 63 outcomes, and further work also needs to establish if these UK data reflect the patient perspective internationally. These themes and subthemes were reviewed alongside summary data generated from a study involving 50 PsA patients in focus groups in Australia, Brazil, France, The Netherlands, Singapore, and the US. The two datasets were largely similar, and combined data from both studies have been taken forward to seek international patient and physician consensus for an updated PsA Core Domain Set [27].

### Strengths and Limitations

The involvement of patient research partners (JL and CB), as well as the use of a maximum variation sampling approach, has increased the likelihood that findings are relevant to a large number of patients with PsA. In addition, the cofacilitators (ED and SH) adopted an inductive approach to data collection and analysis. Therefore, findings reflect the patient perspective on important outcomes, without being heavily influenced by the assumptions of clinicians and researchers. A limitation relates to focus groups as a method of data collection. In a group setting, there is the potential for some participants to feel less able to express their point of view than others; however, there were sufficient focus groups to explore the same topics with different participants. In addition, the cofacilitators intervened to include participants if they perceived an imbalance or dominance of a single viewpoint.

## Conclusion

Qualitative data captured important outcomes of treatment from the perspective of patients with PsA. Over 60 outcomes were identified and grouped into four themes. These highlight the symptoms that most affect patients, the impact these can have on their daily lives, the patients’ anxieties and concerns in relation to pharmacological treatments, and their expectations about benefits and long-term prognosis. There is a need to establish how identified outcomes are represented in existing measures to ensure the inclusion of the patient perspective in future research and clinical practice. Research is also needed to understand patients’ treatment beliefs and the role of the clinical team in communicating treatment-related information.

## References

[1] Lee S, Mendelsohn A, Sarnes E (2010). The burden of psoriatic arthritis: a literature review from a global health systems perspective. P T.

[2] Tillett W, de-Vries C, McHugh NJ (2012). Work disability in psoriatic arthritis: a systematic review. Rheumatology.

[3] Ogdie A, Langan S, Love T (2013). Prevalence and treatment patterns of psoriatic arthritis in the UK. Rheumatology.

[4] Ogdie A, Gelfand J (2015). Clinical risk factors for the development of psoriatic arthritis among patients with psoriasis: a review of available evidence. Curr Rheumatol Rep.

[5] Ramiro S, Smolen J, Landewe R (2016). Pharmacological treatment of psoriatic arthritis: a systematic literature review for the 2015 update of the EULAR recommendations for the management of psoriatic arthritis. Ann Rheum Dis.

[6] Smolen J, Braun J, Dougados M (2014). Treating spondyloarthritis, including ankylosing spondylitis and psoriatic arthritis, to target: recommendations of an international task force. Ann Rheum Dis.

[7] Aletaha D (2016). The many faces of psoriatic arthritis: a challenge to treatment to target?. Rheumatologia.

[8] Tillett W, Adebajo A, Brooke M (2014). Patient involvement in outcome measures for psoriatic arthritis. Curr Rheumatol Rep.

[9] Palominos P, Gaujoux-Viala C, Fautrel B, Dougados M, Gossec L (2012). Clinical outcomes in psoriatic arthritis: a systematic literature review. Arthritis Care Res.

[10] Coates L, Mumtaz A, Helliwell P (2011). Development of a disease severity and responder index for psoriatic arthritis (PsA): report of the OMERACT 10 PsA special interest group. J Rheumatol.

[11] Fitzpatrick R, Davey C, Buxton M, Jones D. Evaluating patient-based outcome measures for use in clinical trials: a review. Health Technol Assess. 1998;2(14):i–iv, 1–74. 9812244

[12] Tillett W, Eder L, Goel N (2015). Enhanced patient involvement and the need to revise the core set: report from the psoriatic arthritis working group at OMERACT 2014. J Rheumatol.

[13] De Wit M, Kirwan J, Tugwell P, et al. Successful stepwise development of patient research partnership: 14 years’ experience of actions and consequences in Outcome Measures in Rheumatology (OMERACT). Patient. Epub 5 Oct 2016. 10.1007/s40271-016-0198-4 PMC5362656 27704486

[14] Gossec L, De Witt M, Kiltz U (2014). A patient-derived and patient-reported outcome measure for assessing psoriatic arthritis: elaboration and preliminary validation of the Psoriatic Arthritis Impact of Disease (PsAID) questionnaire, a 13-country EULAR initiative. J Rheumatol.

[15] Tong A, Sainsbury P, Craig J (2007). Consolidated criteria for reporting qualitative research (COREQ): a 32-item checklist for interviews and focus groups. Int J Qual Health Care.

[16] Krueger R, Casey M (2000). Focus groups: a practical guide for applied research.

[17] Fries J, Spitz P, Kraines R, Holman H (1980). Measurement of patient outcome in arthritis. Arthritis Rheum.

[18] Braun V, Clarke V (2006). Using thematic analysis in psychology. Qual Res Psychol.

[19] Bowen G (2008). Naturalistic inquiry and the saturation concept: a research note. Qual Res.

[20] Kalyoncu U, Ogdie A, Campbell W (2016). Systematic literature review of domains assessed in psoriatic arthritis to inform update of the psoriatic arthritis core domain set. RMD Open.

[21] Hewlett S, Ambler N, Almeida C (2011). Self-management of fatigue in rheumatoid arthritis: a randomised controlled trial of group cognitive-behavioural therapy. Ann Rheum Dis.

[22] Vangeli E, Bakhshi S, Baker A (2015). A systematic review of factors associated with non-adherence to treatment for immune-mediated inflammatory diseases. Adv Ther.

[23] Betteridge N, Boehncke W, Bundy C (2016). Promoting patient-centred care in psoriatic arthritis: a multidisciplinary European perspective on improving the patient experience. J Eur Acad Dermatol Venereol.

[24] Sanderson T, Hewlett S, Flurey C (2011). The impact triad (severity, importance, self-management) as a method of enhancing measurement of personal life impact of rheumatic diseases. J Rheumatol.

[25] Nicklin J, Cramp F, Kirwan J (2010). Collaboration with patients in the design of patient-reported outcome measures: capturing the experience of fatigue in rheumatoid arthritis. Arthritis Care Res.

[26] Nicklin J, Cramp F, Kirwan J (2010). Measuring fatigue in rheumatoid arthritis: a cross-sectional study to evaluate the Bristol Rheumatoid Arthritis Fatigue Multi-Dimensional questionnaire, visual analogue scales, and numerical rating scales. Arthritis Care Res.

[27] Orbai A-M, de Wit M, Mease P, et al. International patient and physician consensus on psoriatic arthritis outcomes for clinical trials. Ann Rheum Dis. Epub 9 Sep 2016. 10.1136/annrheumdis-2016-210242 PMC5344772 27613807

