# Correction to: Important Treatment Outcomes for Patients with Psoriatic Arthritis: A Multisite Qualitative Study

**DOI:** 10.1007/s40271-019-00362-1

**Published:** 2019-03-23

**Authors:** Emma Dures, Sarah Hewlett, Jane Lord, Clive Bowen, Neil McHugh, For the PROMPT Study Group, William Tillett

**Affiliations:** 1 University of the West of England, Bristol, UK; 2 University Hospitals Bristol, Bristol, UK; 3 The Psoriatic Arthritis Support Group (PsAZZ), Bath, UK; 4 University of Bath, Bath, UK; 5 Royal National Hospital for Rheumatic Diseases, Bath, UK; 6 Academic Rheumatology, Bristol Royal Infirmary, Bristol, BS2 8HW UK

## Correction to: Patient (2017) 10:455–462 10.1007/s40271-017-0221-4

The Open Access license, which previously read:

**Open Access** This article is distributed under the terms of the Creative Commons Attribution-NonCommercial 4.0International License (http://creativecommons.org/licenses/by-nc/4.0/), which permits any noncommercial use, distribution, and reproduction in any medium, provided you give appropriate credit to the original author(s) and the source, provide a link to the Creative Commons license, and indicate if changes were made.

Should read:

**Open Access** This article is distributed under the terms of the Creative Commons Attribution 4.0 International License (http://creativecommons.org/licenses/by/4.0/), which permits unrestricted use, distribution, and reproduction in any medium, provided you give appropriate credit to the original author(s) and the source, provide a link to the Creative Commons license, and indicate if changes were made.

The original article has been corrected.

